# Supplementary material for: Unconditionally secured classical cryptography using quantum superposition and unitary transformation
Source: Sci Rep. 2020 Jul 15;10:11687. doi: 10.1038/s41598-020-68038-7 (PMC7363683; doi:10.1038/s41598-020-68038-7)
Supplement: Supplementary file 1 — Supplementary file1 (PDF 388 kb) [file 41598_2020_68038_MOESM1_ESM.pdf]

Supplementary Information for

Unconditionally secured classical cryptography using quantum superposition and unitary transformation

Byoung S. Ham
GIST, S. Korea
bham@gist.ac.kr

Section S1. Proof of unitary matrix of MZI

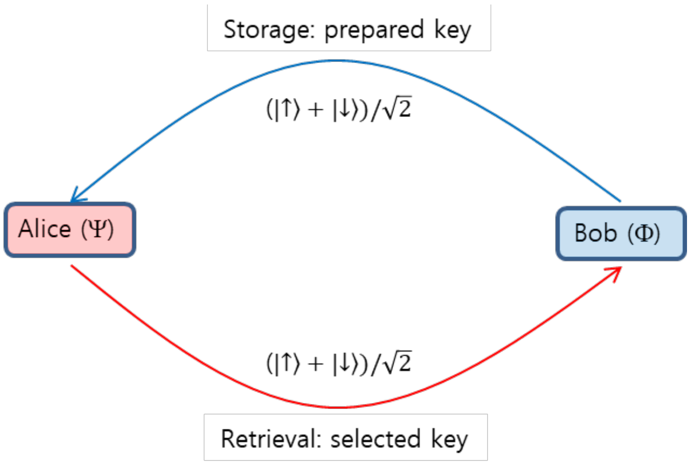

Fig. S1. A schematic of unitary transformation in a round-trip MZI for unconditionally secured classical cryptography. The basis set of $|\uparrow\rangle$ and $|\downarrow\rangle$ represents for any orthogonal relation such as 0 and π in phase or vertical and horizontal in polarization.

Figure S1 is a schematic of the present unconditionally secured classical cryptography (USCC), where the unconditional security is based on deterministic randomness in a round-trip Mach-Zehnder interferometer (MZI) as presented in Fig. 2 with equations (4) and (5) in the main text. Each path in Fig. S1 represents a pair of MZI channels. Compared with quantum cryptography (QKD) based on canonical (non-orthogonal) bases for a key satisfying non-commuting relationship according to quantum mechanics, the unitary transformation in Fig. S1 is for non-canonical (orthogonal bases) bases in a pair of classical channels, where the deterministic randomness of USCC results from the MZI path superposition and its double unitary transformation. Here, the double unitary transformation represents time reversal process retrieving eigenvalues. The randomness results from the indistinguishability of the orthogonal bits in the superposed path of MZI. This is the heart of deterministic randomness. The path superposition of MZI is of course replaced by basis superposition as shown. By the way, the no-cloning theorem is the direct result of canonical variable-based Heisenberg's uncertainty principle in quantum mechanics and becomes the bedrock of the unconditional security in QKD. Likewise, the deterministic randomness resulting from the MZI path superposition and unitary transformation corresponds to the no-cloning theorem of QKD and becomes the heart of USCC.

In general unitary transformation supports the basic mechanism of an optical (quantum) memory. In Fig. S1, binary bases (orthogonal phases) are used for the deterministic randomness, where the round tripped basis is supposed to be either itself or the other depending on the basis choice by the other party (Alice). To work with the deterministic randomness Bob prepares random basis, and it to Alice. This a typical storage process in an optical memory. Then, Alice reads it out with her random basis choice. If it is the same as what Bob chose it results in the same basis (identity) to Bob when the signal is returned. If Alice chooses the opposite basis, it results in the opposite basis (inversion) to Bob, too. Such a result is automatically determined by the MZI directionality to both Alice and Bob. Here, the inversion stands for basis swapping in the binary system. Unlike QKD requiring a sifting process via post-measurements, the deterministic randomness in USCC is highly efficient due to its automatic process as discussed in Fig. 1. Moreover, USCC works for both quantum and classical bases, where MZI physics does not discriminates both. For the classical one, thus, USCC is

compatible with current fiber-optic communication systems.

At each path in Fig. S1, Eve (MZI channel eavesdropper) has a 50% chance of eavesdropping like coin tossing due to the MZI path superposition, resulting in measurement indistinguishability as in the Young's double-slit experiment. The 50% eavesdropping chance is simply due to the use of equally weighted two-orthogonal bases. If N orthogonal basis pairs are involved for N paired remotely separated parties, USCC can also be applicable to semi-continuous variable version (discussed elsewhere). Thus, the novelty of the present USCC is that the quantum nature of the unconditional security in QKD can be achieved in a classical regime by using orthogonal bases. The potential of USCC is the application to the one-time-pad cryptography owing to ultrafast key distribution rate based on the classical compatibility with current optical systems.

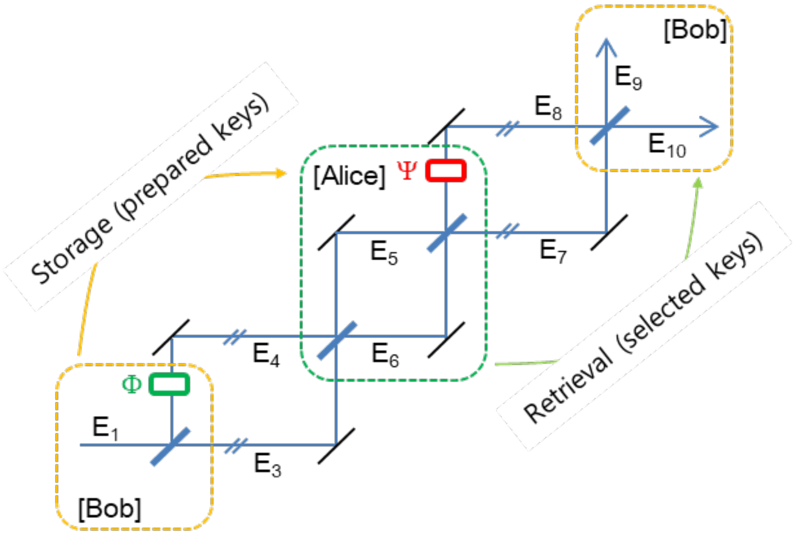

Fig. S2. The unfolded scheme of Fig. S1 (see also Fig. 2).

Figure S2 shows an unfolded scheme of Fig. 2 (or Fig. S1). Each channel of Fig. S1 is now represented by two MZI paths of E_3 & E_4 or E_7 & E_8. The MZI path superposition results in the directional determinacy in the output fringe (interference) of E_5 & E_6 and E_9 & E_{10}. The random choice (shuffling) of ψ keeps its determinacy to both parties but randomness to an eavesdropper. The possible combinations with binary bases are four as shown in Table 1(c). With the binary basis set, the determinacy through MZI is either identity inversion. The binary set is represented by orthogonal phase, 0 and π. Using matrix representation with the beam splitter (BS) and phase rotation (Φ), MZI physics for unitary transformation is as follows:

$$\begin{bmatrix} E_7 \\ E_8 \end{bmatrix} = [BS][\Phi]_{\psi}[BS][BS][\Phi]_{\varphi}[BS]\begin{bmatrix} E_1 \\ E_2 \end{bmatrix},$$

$$= \frac{1}{4}\begin{bmatrix} 1 & i \\ i & 1 \end{bmatrix}\begin{bmatrix} 1 & 0 \\ 0 & e^{i\psi} \end{bmatrix}\begin{bmatrix} 1 & i \\ i & 1 \end{bmatrix}\begin{bmatrix} 1 & i \\ i & 1 \end{bmatrix}\begin{bmatrix} 1 & 0 \\ 0 & e^{i\varphi} \end{bmatrix}\begin{bmatrix} 1 & i \\ i & 1 \end{bmatrix}\begin{bmatrix} E_1 \\ 0 \end{bmatrix}. \quad \text{(S1)}$$

For the same basis choice by Alice ($\psi = \varphi$) satisfying the identity relation, the output of $\begin{bmatrix} E_7 \\ E_8 \end{bmatrix}$ is represented by:

$$\begin{bmatrix} E_7 \\ E_8 \end{bmatrix} = [MZ]_\varphi^2 \begin{bmatrix} E_1 \\ 0 \end{bmatrix}$$ as shown in equation (3) in the main text,

where $[MZ]_\varphi = [BS][\Phi][BS] = \frac{1}{2}\begin{bmatrix} (1-e^{i\varphi}) & i(1+e^{i\varphi}) \\ i(1+e^{i\varphi}) & -(1-e^{i\varphi}) \end{bmatrix}$ and $[MZ]_\varphi^2 = (-e^{i\varphi})\begin{bmatrix} 1 & 0 \\ 0 & 1 \end{bmatrix}$. Thus, E_7 and E_8 have the same value regardless of the basis choice, resulting in the measurement randomness to Eve.

Now, let us define $A \equiv [MZ]_\varphi$. Then, $A^\dagger$ is represented by:

$$A^\dagger = \frac{1}{2}\begin{bmatrix} (1-e^{-i\varphi}) & -i(1+e^{-i\varphi}) \\ -i(1+e^{-i\varphi}) & -(1-e^{-i\varphi}) \end{bmatrix}. \tag{S2}$$

If $A^\dagger A = I$, A is proved to be unitary satisfying a reversible process in Fig. S1:

$$A^\dagger A = \frac{1}{4}\begin{bmatrix} (1-e^{-i\varphi}) & -i(1+e^{-i\varphi}) \\ -i(1+e^{-i\varphi}) & -(1-e^{-i\varphi}) \end{bmatrix}\begin{bmatrix} (1-e^{i\varphi}) & i(1+e^{i\varphi}) \\ i(1+e^{i\varphi}) & -(1-e^{i\varphi}) \end{bmatrix}$$

$$=\frac{1}{4}\begin{bmatrix} (1-e^{-i\varphi})(1-e^{i\varphi}) + (1+e^{-i\varphi})(1+e^{i\varphi}) & i(1-e^{-i\varphi})(1+e^{i\varphi}) + i(1+e^{-i\varphi})(1-e^{i\varphi}) \\ -i(1+e^{-i\varphi})(1-e^{i\varphi}) - i(1-e^{-i\varphi})(1+e^{i\varphi}) & (1+e^{-i\varphi})(1+e^{i\varphi}) + (1-e^{-i\varphi})(1-e^{i\varphi}) \end{bmatrix}$$

$$=\frac{1}{4}\begin{bmatrix} 1+1-e^{i\varphi}-e^{-i\varphi}+1+1+e^{i\varphi}+e^{-i\varphi} & i(1-1+e^{i\varphi}-e^{-i\varphi}+1-1+e^{-i\varphi}-e^{i\varphi}) \\ -i(1-1+e^{-i\varphi}-e^{i\varphi}+1-1+e^{i\varphi}-e^{-i\varphi}) & 1+1+e^{i\varphi}+e^{-i\varphi}+1+1-e^{i\varphi}-e^{-i\varphi} \end{bmatrix}$$

$$=\begin{bmatrix} 1 & 0 \\ 0 & 1 \end{bmatrix}$$

$$=I. \tag{S3}$$

Because the global phase of $[MZ]_\varphi^2$ has nothing to do with the unitary transformation, the MZI matrix in Fig. S2 is proved to be unitary. Thus, the scheme of the round-trip MZI in Fig. S2 (also Fig. 2) represents the time reversibility such as in an optical memory. This is the novelty of USCC representing deterministic randomness. The followings are additional information for equation (S3):

(i) For $\varphi = 0$, the matrix representation for the outputs of BS in Fig. S2 is as follows:

$$\begin{bmatrix} E_3 \\ E_4 \end{bmatrix} = [\Phi]_{\varphi=0}[BS]\begin{bmatrix} E_1 \\ 0 \end{bmatrix} = \frac{1}{\sqrt{2}}\begin{bmatrix} 1 & 0 \\ 0 & 1 \end{bmatrix}\begin{bmatrix} 1 & i \\ i & 1 \end{bmatrix}\begin{bmatrix} E_1 \\ 0 \end{bmatrix} = \frac{1}{\sqrt{2}}\begin{bmatrix} E_1 \\ iE_1 \end{bmatrix};$$

$$\begin{bmatrix} E_5 \\ E_6 \end{bmatrix} = [BS]\begin{bmatrix} E_3 \\ E_4 \end{bmatrix} = \frac{1}{2}\begin{bmatrix} 1 & i \\ i & 1 \end{bmatrix}\begin{bmatrix} E_1 \\ iE_1 \end{bmatrix} = i\begin{bmatrix} 0 \\ E_1 \end{bmatrix};$$

$$\begin{bmatrix} E_7 \\ E_8 \end{bmatrix} = [\Phi]_{\delta=0}[BS]\begin{bmatrix} E_5 \\ E_6 \end{bmatrix} = \frac{i}{\sqrt{2}}\begin{bmatrix} 1 & 0 \\ 0 & 1 \end{bmatrix}\begin{bmatrix} 1 & i \\ i & 1 \end{bmatrix}\begin{bmatrix} 0 \\ E_1 \end{bmatrix} = \frac{1}{\sqrt{2}}\begin{bmatrix} -E_1 \\ iE_1 \end{bmatrix};$$

$$\begin{bmatrix} E_9 \\ E_{10} \end{bmatrix} = [BS]\begin{bmatrix} E_7 \\ E_8 \end{bmatrix} = \frac{1}{2}\begin{bmatrix} 1 & i \\ i & 1 \end{bmatrix}\begin{bmatrix} -E_1 \\ iE_1 \end{bmatrix} = (-1)\begin{bmatrix} E_1 \\ 0 \end{bmatrix}.$$

(ii) For $\varphi = \pi$, the matrix representation for the outputs of BS in Fig. S2 is as follows:

$$\begin{bmatrix} E_3 \\ E_4 \end{bmatrix} = [\Phi]_{\varphi=\pi}[BS]\begin{bmatrix} E_1 \\ 0 \end{bmatrix} = \frac{1}{\sqrt{2}}\begin{bmatrix} 1 & 0 \\ 0 & -1 \end{bmatrix}\begin{bmatrix} 1 & i \\ i & 1 \end{bmatrix}\begin{bmatrix} E_1 \\ 0 \end{bmatrix} = \frac{1}{\sqrt{2}}\begin{bmatrix} E_1 \\ -iE_1 \end{bmatrix};$$

$$\begin{bmatrix} E_5 \\ E_6 \end{bmatrix} = [BS]\begin{bmatrix} E_3 \\ E_4 \end{bmatrix} = \frac{1}{2}\begin{bmatrix} 1 & i \\ i & 1 \end{bmatrix}\begin{bmatrix} E_1 \\ -iE_1 \end{bmatrix} = \begin{bmatrix} E_1 \\ 0 \end{bmatrix};$$

$$\begin{bmatrix} E_7 \\ E_8 \end{bmatrix} = [\Phi]_{\delta=\pi}[BS]\begin{bmatrix} E_5 \\ E_6 \end{bmatrix} = \frac{1}{\sqrt{2}}\begin{bmatrix} 1 & 0 \\ 0 & -1 \end{bmatrix}\begin{bmatrix} 1 & i \\ i & 1 \end{bmatrix}\begin{bmatrix} E_1 \\ 0 \end{bmatrix} = \frac{1}{\sqrt{2}}\begin{bmatrix} E_1 \\ -iE_1 \end{bmatrix};$$

$$\begin{bmatrix} E_9 \\ E_{10} \end{bmatrix} = [BS]\begin{bmatrix} E_7 \\ E_8 \end{bmatrix} = \frac{1}{2}\begin{bmatrix} 1 & i \\ i & 1 \end{bmatrix}\begin{bmatrix} E_1 \\ -iE_1 \end{bmatrix} = \begin{bmatrix} E_1 \\ 0 \end{bmatrix}.$$

As shown above, Bob’s detection for the round-trip light through the same MZI paths is identical regardless of basis choice, representing the unitary transformation. Thus, Alice’s role is clear whether to keep the identity the same or opposite.

Table S1. A comparison chart between QKD and USCC

**Parameter**	**QKD**	**USCC**	
Unconditional security	Uncertainty principle	Superposition (MZI paths)	With randomness
Key distribution	One way (sifting)	Round trip (unitary)	Randomness
Key rate	$<10^6$	$>10^9$	
Basis	Non-orthogonal (canonical)	Orthogonal (non-canonical)	Unitary transformation
Duplication (definite or specific)	No (no-cloning theorem)	No (MZI indistinguishability)	Unconditional security
Duplication (blind)	No (no-cloning theorem)	Yes (classical)	Amplifier compatibility
Randomness	Post-selection	Pre-selection	Sifting vs. determinacy
Network compatibility (classical)	No	Yes	Coherence optics (bright light)
Network compatibility (quantum)	Yes	Yes	Incoherence optics (single photon)
Long distance transmission	No	Yes	Blind duplication
OTP compatibility	No (quantum loopholes)	Yes	One-Time-Pad applications
Photon feature	Non-classical	Classical (including single photon)	

Table S1 is a comparison chart between USCC and QKD. Because USCC is based on the beam-splitter optics, it covers both coherence (bright light) and incoherence (single photon) optics. Thus, USCC includes a specific QKD based on single photons such as BB84. The fundamental difference between USCC and QKD is the origin of unconditional security. Unlike QKD based on on-cloning theorem of quantum mechanics and canonical bases, USCC is based on quantum superposition of two transmission paths for non-canonical bases of key carriers. As explained in Figs. S1 and S2, the unconditional security is resulted from random basis shuffling by one party for the prepared one by another party. Thus, USCC is compatible with classical optics in principle. Obviously the territory of USCC can be expanded from the binary system to N-ary one (many bases).

Section S2. Key distribution procedure of USCC without sifting (post-measurement)

The USCC key distribution procedure without sifting results in 100% key rate due to the MZI determinacy if there is no network error (red). However, each key must follow the network reset protocol mentioned in Discussion to avoid of classical memory-based attack.

Table S2. OKD procedure without sifting for Fig. 2.

Party	Sequence	Order	1	2	3	4	5	6	7	8	9	10	set
Alice	3	V_A	1	−1	−1	1	−1	1	1	−0.5*	1	−1	
	3	Copy x: y	0	1	1	0	1	0	0	−0.5	0	1	$\{y\}$
	4	ψ	0	0	π	0	π	0	π	π	π	π	
	5	z(ψ)	0	0	1	0	1	0	1	1	1	1	$\{z\}$
	7	Final key	**1**	**0**	**1**	**1**	**1**	**D**	**0**	**D****	**0**	**1**	$\{m\}$
Bob	1	φ	0	π	π	0	π	0	0	π	0	π	
	2	Prepared key: x(φ)	0	1	1	0	1	0	0	1	0	1	$\{x\}$
	6	V_B	−1	1	−1	−1	1	−0.8*	1	−1	1	−1	
	7	Final key: m	**1**	**0**	**1**	**1**	**1**	**D****	**0**	**D**	**0**	**1**	$\{m\}$

*The numbers in red refer to network errors or eavesdropping.
**Only error bits are announced publically to discard the corresponding bit from the final key set $\{m\}$. Here, D represents an error bit and can be set to any number, e.g., D=9 for a computing algorithm.
$V_A = V_{5,6};\ V_B = V_{9,10};\ V_{ij} = \frac{I_j - I_i}{I_j + I_i}$

Sequence:

1. Bob randomly selects his phase $\varphi \in \{0, \pi\}$ to create a φ −controlled coherent light pulse via the phase shifter Φ and sends it to Alice. Here, the φ −controlled light can be an N-bit chain.
2. Bob converts his chosen φ into a key in $\{x\}$ for a record: $x \in \{0,1\}$, where $x = 0$ if $\varphi = 0$, and $x = 1$ if $\varphi = \pi$.
3. Alice measures her detectors A_1 and A_2 for visibility V_A to copy Bob's key $\{x\}$ in $\{y\}$: $y = 0$ if $V_A = 1$; $y = 1$ if $V_A = -1$; $y = V_A$ if $V_A \neq \pm 1$; $\{y\} = \{x\}$, except for $V_A \neq \pm 1$. Here, $V_A \neq \pm 1$ stands for an error due to eavesdropping or network problem: see the red number in Table S2.
4. Alice randomly selects her phase $\psi \in \{0, \pi\}$ to create a ψ −controlled light pulse via the phase shifter Ψ and sends it back to Bob. Here, the ψ −phase control is performed on the reflected φ −controlled light pulse(s).
5. Alice converts her chosen ψ into a key in $\{z\}$ for a record: $z \in \{0,1\}$, where $z = 0$ if $\psi = 0$, and $z = 1$ if $\psi = \pi$.
6. Bob measures his detectors B_3 and B_4 for visibility V_B: $w = x$ if $V_B = -1$; w=D if $V_B = 1$; $w = V_B$ if $V_B \neq \pm 1$. Here, $V_B \neq \pm 1$ stands for an error due to eavesdropping or network problem.
7. Alice and Bob publically announce their error bits (D) for $V_B \neq \pm 1$ and $V_B \neq \pm 1$. Alice and Bob now share the same key $\{m\}$. For Alice, $m = (y + z) \oplus 1$ at modulus 2. For Bob, m=1 $V_B = -1$ and m=0 if $V_B = 1$; D if $V_A \neq \pm 1$ or $V_B \neq \pm 1$.

Section S3. Example of the memory-based attack

For a given n-bit long key whose basis is binary, the maximum number of representation is 2^n. For n=126, thus, the total number of representation is $2^{128} = 3.4\text{x}10^{38}$. Recalling the universe age is 1.38×10^{10} years or 4.35×10^{17} seconds, the eavesdropping chance for the 128-bit long key by using the most powerful supercomputer (IBM Summit) whose performance is 1.43×10^{17} flops/s is as follows:

$$\eta = \frac{6.2\times10^{34}}{3.4\times10^{38}} = 1.8 \times 10^{-4},$$

where 6.2×10^{34} is from the universe age $(1.43 \times 10^{17})(4.35 \times 10^{17})$.

Even with a personal computer whose operating system is 64-bit based, the brute force attack for the randomness in USCC takes more than 100 seconds: $2^{64} = 1.8 \times 10^{19}$. This means that the proposed USCC is effective even with personal computers for the applications of the one-time-pad cryptography, where the key never be reused and the key distribution speed is a few microseconds limited by the transmission distance rather than optical devices or CPU clock speed higher than 1 GHz. The flight time Δt of a light pulse for a 10 km optical fiber is as follows:

$$\Delta t = \text{n}\frac{1\times10^{4}}{3\times10^{8}} = 5 \times 10^{-5} \text{ (s)},$$

where n is the refractive index of the optical fiber, n~1.5. This channel-bandwidth bottleneck can also be solved by multi-channel configuration in current fiber-optic communications networks.

Section S4. Network initialization

As mentioned in the main text, Alice has two options of added δ setting for her phase shifter Ψ either she gets a constructive or destructive fringe. Table S3 is the opposite case of Table 3 in the main text for the network initialization, where Alice sets for maxima with δ as destructive interference in the network reset process. The eavesdropper Eve can do the same job as Alice does. However, Eve's best chance is 50% of what Alice does. The network initialization results in bit-by-bit randomness preventing Eve from the memory-based attack.

Table S3. Network initialization.

Party	Sequence	Order (N)	1	2	3	4	5	6	7	8	9	10
Alice	1	Ψ	δ	δ	δ+π	δ	δ+π	δ+π	δ	δ+π	δ	δ+π
	3	V_A	1	−1	−1	1	−1	1	1	1	−1	1
Bob	2	φ	0	π	π	0	π	0	0	0	π	0
	4	V_B	1	−1	1	1	1	−1	1	−1	−1	−1
		Correctness	X	O	X	X	X	O	X	O	O	O

*$V_A = V_{5,6}$; $V_B = V_{9,10}$

*Table S2 is for π–added δ.

Sequence:

1. Initially Alice resets the MZI network by adjusting a path length and scan δ added to her phase basis $\psi \in \{0, \pi\}$ until she gets $V_A = \pm 1$ for the same test keys provided by Bob.
2. Bob randomly selects $\varphi \in \{0, \pi\}$, encodes his optical key with φ, and sends it to Alice.
3. Alice measures V_A for maxima with δ– added ψ and publically announces the result.
4. Bob measures his V_B and publically announces whether it is correct (O) or not (X).
5. Alice resets her phase basis $\psi \in \{0, \pi\}$ to either $\psi \in \{\delta, \pi + \delta\}$ or $\psi \in \{-\delta, \pi - \delta\}$ depending on the correctness.
6. The sequence 1~5 is repeated for the next order (column). Here the next order is for either each block $\{m\}$ with sifting or each bit (column) without sifting in Table 2.

[Network initialization flow chart for each key in Table S3]

The following flow chart is for each sequence in Table S3, where each bit of an N-sized key is repeated sequentially. By doing this, Eve's memory-based attack is not effective anymore.

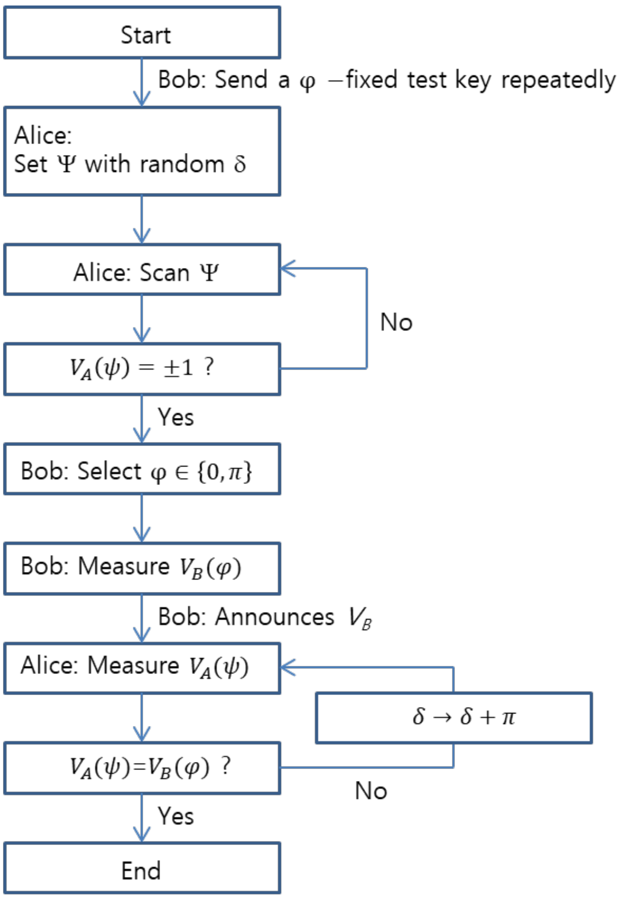

Section S5. Applications of USCC

Figure S3 shows an application of the round-trip MZI-based USCC in Fig. 2 for multi-core fiber-optic networks. Although double single-core fibers can replace the multi-core fiber without losing network stability [23], the multi-core fiber may give a technical simplicity due to the frozen relative phase drift.

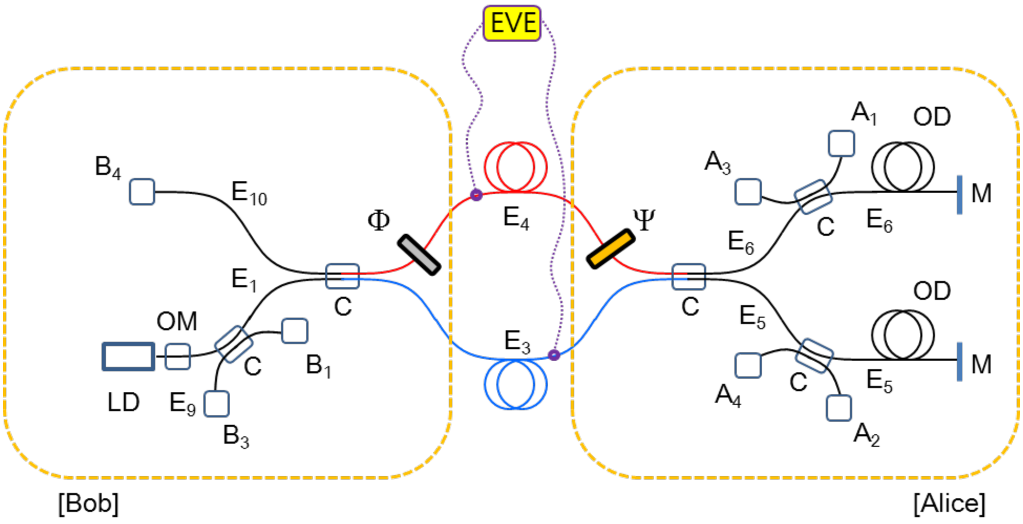

Fig. S3. A schematic of optical fiber-based USCC. L, Laser; OM, optical modulator; OD, optical delay; A_i, detector at Alice side; B_i, detectors at Bob's side; M, mirror; Φ, phase controller at Bob's side; Ψ, phase controller at Alice's side. E_i, coherent light pulse. C, 50/50 fiber coupler.
